# Quadricuspid Right Atrioventricular Valve: A Cadaveric Case Report

**DOI:** 10.7759/cureus.92930

**Published:** 2025-09-22

**Authors:** Tanner Buckway, Mallory Weed, Alejandra Tobon, Amberly Reynolds

**Affiliations:** 1 Department of Anatomical Sciences, Rocky Vista University College of Osteopathic Medicine, Ivins, USA

**Keywords:** anatomical variation, cadaveric case report, cardiac anatomy, embryology, gross anatomy

## Abstract

Quadricuspid heart valve anomalies are rare congenital malformations, typically affecting the aortic valve; involvement of the right atrioventricular (tricuspid) valve is exceedingly uncommon. Embryologically, the tricuspid valve forms through complex fusion and delamination processes involving the endocardial cushions.

This case describes a quadricuspid right atrioventricular valve identified during routine cadaveric dissection in an elderly male. Gross examination revealed four distinct cusps and an accessory papillary muscle, along with right atrial dilation, near-complete atrophy of the right auricle, right ventricular hypertrophy, and evidence of poor valve coaptation. These findings, combined with signs of cardiomegaly, suggest significant valvular dysfunction during life, likely contributing to chronic tricuspid regurgitation. During the gross examination, valvular distortion was noted, highlighting the potential for secondary degenerative changes superimposed upon congenital structural anomalies, particularly in the elderly.

Recognition of such anomalies is essential, as they may predispose individuals to valvular incompetence, right-sided heart dysfunction, and other clinical complications. Early identification and characterization of these rare malformations may influence clinical management and surgical decision-making.

## Introduction

Cardiac valves ensure unidirectional blood flow through the heart by opening and closing in response to pressure gradients. Each valve is composed of cusps, thin yet durable flaps of tissue that form a tight seal during closure. The atrioventricular valves, including the tricuspid and mitral valves, are anchored by chordae tendineae--fibrous cords that attach the cusps to papillary muscles within the ventricles [1]. During systole, contraction of the papillary muscles maintains tension on the chordae tendineae, preventing cusp prolapse into the atria and ensuring efficient forward flow. This coordinated interaction between cusps, chordae tendineae, and papillary muscles is critical for maintaining effective cardiac function [2].

The development of the atrioventricular valve is a complex process involving the fusion and delamination of endocardial cushions between the atrium and ventricle. Around the fifth to eighth weeks of gestation, the atrioventricular canal is partitioned by the fusion of the superior and inferior endocardial cushions [3]. This process results in the formation of the septal, anterior, and posterior leaflets of the tricuspid valve or the right atrioventricular valve [4].

Anatomical variation in the right ventricle has shown bifurcated (15.38-53.33%) and trifurcated (5.76-35.86%) papillary muscles and multiple chordae tendineae attachment points [5]. Quadricuspid morphology in the right atrioventricular valve is extremely uncommon, and the presence of an additional papillary muscle for the fourth cusp is even rarer, with few cases documented in the literature [6]. It has been suggested that the prevalence of right quadricuspid atrioventricular valves is less than 0.01%, making it a particularly rare congenital anomaly [7]. Such anomalies are often incidental findings during autopsy or cardiac imaging and can sometimes lead to tricuspid regurgitation or other functional impairments [8].

This report details a rare quadricuspid right atrioventricular valve identified during routine cadaveric dissection, along with its anatomical, embryological, and clinical significance.

## Case presentation

**Figure 1:**
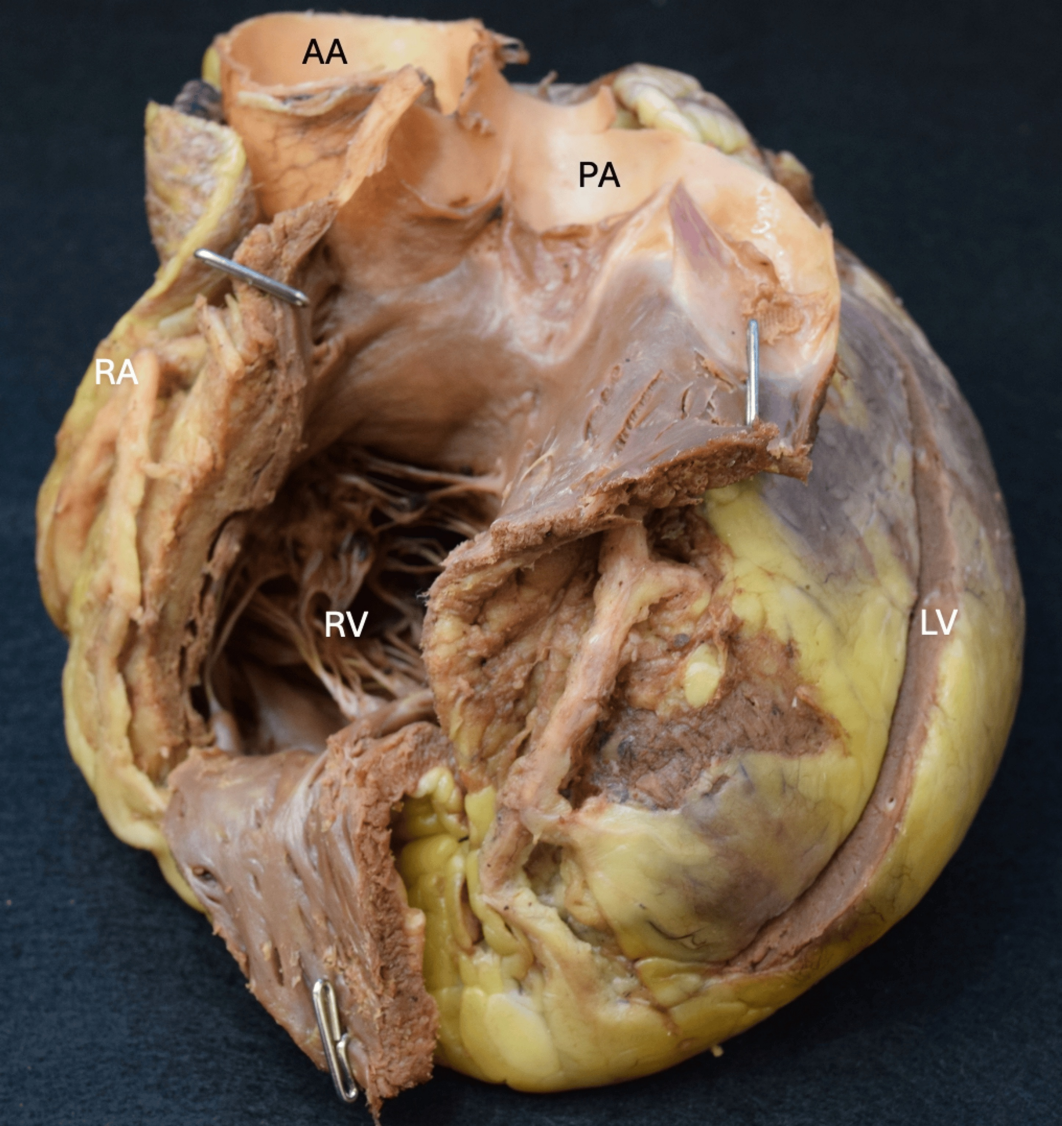
Overview of the cardiac shape with potential cardiomegaly and a cardiothoracic ratio of 0.52 AA: Ascending aorta, PA: Pulmonary artery, RA: Right atrium, RV: Right ventricle, LV: Left ventricle

**Figure 2:**
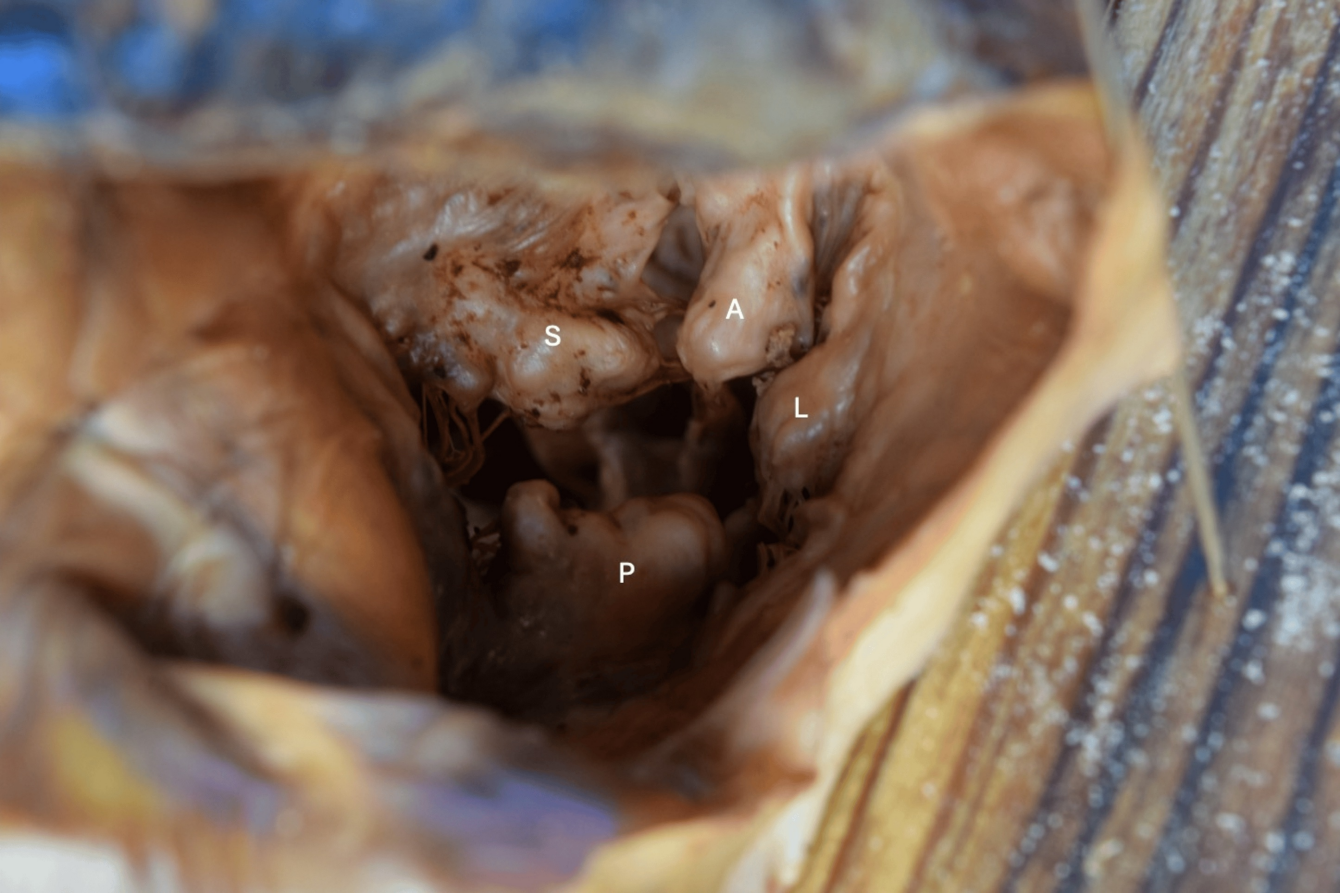
Right atrioventricular valve with abnormal architecture and number of cusps S: Septal cusp, A: Anterior cusp, P: Posterior cusp, L: Lateral cusp

**Figure 3:**
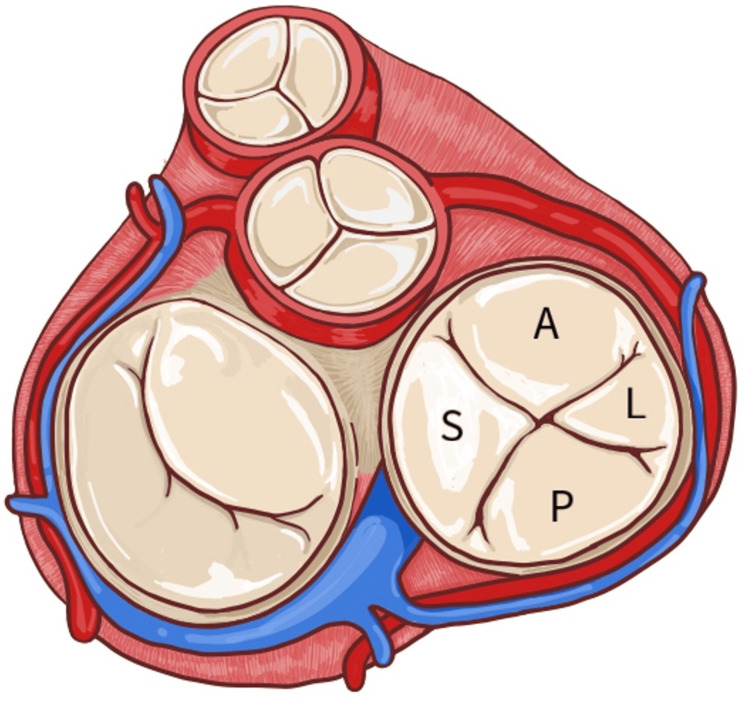
Anatomical drawing of the right atrioventricular valve with approximate cusp proportions A: Anterior cusp, L: Lateral cusp, P: Posterior cusp, S: Septal cusp Original drawing created by Alejandra Tobon, Second Year Osteopathic Medical Student.

**Figure 4:**
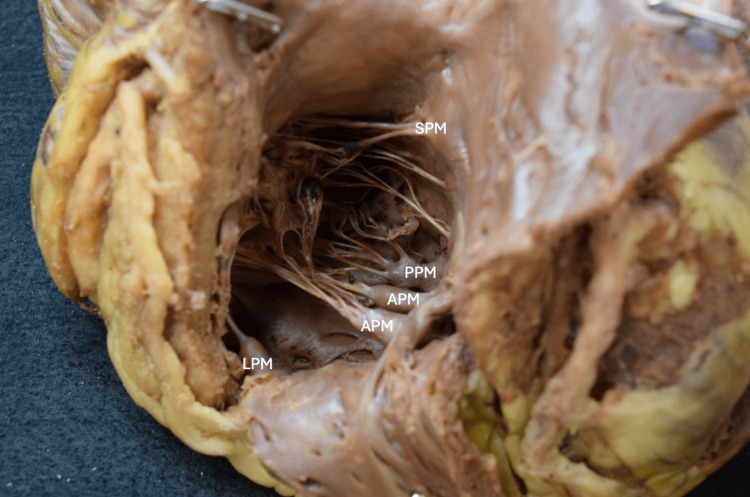
The right ventricle with walls reflected reveals papillary muscles. The accessory papillary muscle, labeled as the lateral papillary muscle, is located on the superolateral aspect of the right ventricular wall. The anterior papillary muscle contained two heads. SPM: Septal papillary muscle, PPM: Posterior papillary muscle, APM: Anterior papillary muscle, LPM: Lateral papillary muscle

**Figure 5:**
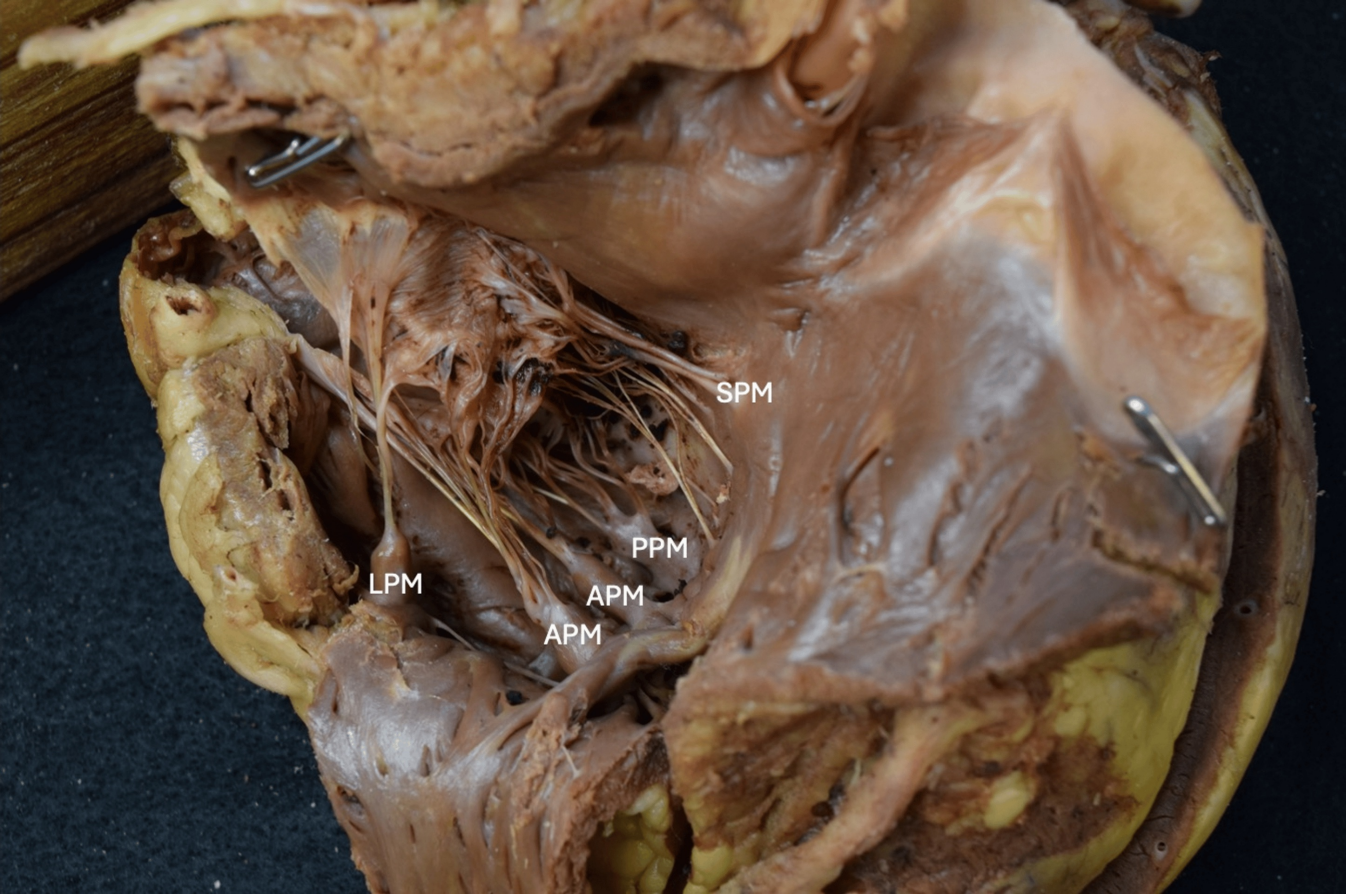
The right ventricle with walls reflected to appreciate the attachment points of the chordae tendineae to papillary muscles and the associated cusps. SPM: Septal papillary muscle, PPM: Posterior papillary muscle, APM: Anterior papillary muscle, LPM: Lateral papillary muscle

During the routine anatomical dissection of an elderly Caucasian male cadaver, with the cause of death of coronary artery disease, hypertension, and hyperlipidemia, an anomaly was observed in the right atrioventricular valve (Figure 1). Upon inspection, the valve exhibited four distinct cusps of varying size and shape (Figures 2, 3). The cusps were located in their named positions: anterior, posterior, septal, and lateral, with the anterior cusp being slightly smaller than the septal and posterior cusps. The lateral cusp was found on the lateral side of the right atrioventricular valve and was the smallest of the four cusps. An extranumerary papillary muscle was found situated in the right ventricle. The anterior papillary muscle (APM) consisted of two heads. The septal papillary muscle (SPM) and posterior papillary muscle (PPM) were grossly normal. The accessory (lateral) papillary muscle was attached to the superolateral wall of the right ventricle (Figure 4). The chordae tendineae associated with the accessory papillary muscle were found to be attached to an accessory lateral cusp (Figure 5).

The aortic, pulmonic, and mitral valves appeared normal, each with the appropriate number of cusps and without evidence of poor coaptation. The mitral valve also demonstrated the expected complement of papillary muscles with intact chordal support.

In contrast, the cadaver showed evidence of right-sided cardiac enlargement and signs of poor right atrioventricular valvular coaptation, suggesting the valve likely experienced functional impairment during life.

Gross examination of the right side of the heart revealed several secondary structural changes suggestive of chronic valvular insufficiency. These included marked dilation of the right atrium with near-complete atrophy of the right auricle, findings consistent with chronic volume overload. The heart occupied a disproportionately large portion of the thoracic cavity relative to expected norms, and preliminary measurements indicated an increased cardiothoracic ratio of 0.52, 12 cm/23 cm, consistent with cardiomegaly. Gross inspection further revealed regions of leaflet thickening and redundancy.

## Discussion

Quadricuspid atrioventricular valves are thought to arise from disruptions in normal valvulogenesis, specifically during the formation and remodeling of the endocardial cushions. Aberrant partitioning, incomplete reabsorption, or abnormal fusion of these cushions may lead to the development of an additional cusp [9]. If the cushions fail to merge properly, additional cusps may form, as seen in this case. Such variations typically affect the aortic valve rather than the tricuspid valve, making this finding particularly rare [10]. The presence of an additional papillary muscle suggests compensatory development to maintain chordal support for the fourth cusp. This muscle was likely congenitally derived during the formation of the right atrioventricular valve.

Functionally, quadricuspid valves may lead to valvular dysfunction due to incomplete coaptation of the leaflets, resulting in regurgitation. In this case, the right-sided cardiac enlargement and valve architecture support the hypothesis that the anomaly contributed to functional impairment. With advancing age or increased pressure in the right side of the heart, such structural variations can exacerbate valve incompetence, potentially leading to heart failure [8].

Notably, congenital anomalies, such as a quadricuspid morphology, may predispose the valve to secondary degenerative changes over time. Myxomatous changes, characterized by disruption of the normal collagen framework, accumulation of proteoglycans, and fragmentation of elastic fibers, were evident and are known to compromise the structural integrity of valve tissue [11,12]. These alterations predispose valves to prolapse, incomplete closure, and regurgitation, particularly in elderly individuals, and may have exacerbated the insufficiency observed in our patient.

Furthermore, myxomatous degeneration has been linked to increased mechanical stress on valve leaflets [11,13], which in this case, may have been compounded by the abnormal stress distribution across four asymmetric cusps. This interplay between congenital morphology and acquired degeneration could have accelerated valvular dysfunction and contributed to the chamber enlargement and auricular atrophy noted at autopsy. The presence of these degenerative changes also suggests increased susceptibility to future endocardial complications, including progressive regurgitation and vegetation formation. Collectively, the structural abnormalities and degenerative remodeling provide compelling morphological evidence of right-sided heart dysfunction during life.

Quadricuspid valves have been described in prior reports, but these cases have almost exclusively involved the aortic valve rather than the right atrioventricular valve [14]. The present case, therefore, represents a particularly uncommon manifestation. In addition, the presence of an accessory papillary muscle distinguishes this specimen, which has not been widely emphasized in the literature.

A limitation of this report is that the findings are based solely on cadaveric examination. Consequently, there is no accompanying information regarding the individual’s clinical presentation, past medical history, physical exam findings, or imaging studies related to the valve abnormality. Without this data, it is difficult to connect the structural observations to possible manifestations in life, which narrows the ability to assess functional significance or diagnostic considerations in a clinical setting.

Preoperative imaging, such as echocardiography or cardiac magnetic resonance imaging, can aid in the diagnosis of these anomalies. While many cases remain asymptomatic, identification is crucial for patients undergoing cardiac procedures to prevent complications.

## Conclusions

This case report describes a rare quadricuspid right atrioventricular valve with four distinct cusps and an accessory papillary muscle, contributing to functional impairment and right-sided heart enlargement. Embryologically, this anomaly likely resulted from aberrant partitioning and fusion of the endocardial cushions during valve morphogenesis. Although quadricuspid valves are often asymptomatic, they can predispose individuals to tricuspid regurgitation, vegetations, and right-sided heart dysfunction, particularly under conditions of elevated pressure.

Recognition of such rare anomalies is essential for both anatomical education and clinical practice, as they may influence surgical planning and patient management. Further studies are warranted to better understand the incidence, clinical implications, and optimal management of quadricuspid atrioventricular valves.
